# Can a Sixpenny Doctor Be Honest?

**Published:** 1891-07-18

**Authors:** 


### The Hospital

A Weekly Journal of

Science, Medicine, Nursing, and Philanthropy.

*For Hospitals, Asylums, Infirmaries, Dispensaries, Medical Practitioners, Students, Nurses, and the Charitable Public.*

Vol. X.—No. 251.] [July 18, 1891.

### Can a Sixpenny Doctor be Honest?

A week or two ago we wrote a brief article, sharply denouncing certain doctors who have medical clubs, by means of which they profess to treat children medically and to supply them with medicine, for sixpence a quarter. A correspondent in the *Provincial Medical Journal* waxes indignant and eloquent over our plainness of speech. He confesses himself a sixpenny doctor. The confession is somewhat of a surprise to us, because, notwithstanding our article we hoped we might have been misinformed as to the facts, and also because as personally representing qualified medical practice, we are ashamed that there is a single practitioner who can be brought to confess himself a sixpenny doctor, and who has the temerity to defend his position. The medical man in question states that the vicar of his parish has an income of about £70 a year, and he advances this as a justification of his own sixpence a quarter method. The gentleman has evidently studied logic: he is firmly convinced that two wrongs make a right; or at least that two absurdities make one rationality. What a happy man he must be!

It is pretty clear that sixpenny doctors feel sore when they are touched; their withers are not unwrung. That is a hopeful sign. It is quite certain, too, that orthodox practitioners are deeply moved about the subject; practitioners who would rather have no fee at all than descend below the humble half-crown. For did not even the village apothecary think somewhat meanly of the half-crown in those far distant times when James the First was writing his famous "Counterblast"?

The whole question of medical remuneration is urgently in need of discussion; and it ought to be discussed and re-discussed until reasonable practices are arrived at. Both medical men and the public should be aware that the question is not merely one of larger or smaller fees to particular individuals, but of the status, education, and capacity of the medical profession as a whole. To take a parallel case: The newspapers of the very day on which this article is written are loudly complaining that men cannot be found to cultivate the soil of our own country. What is the reason? Farmers have persistently lowered the wages of their workmen for several generations. The result is that all the men of sound bodily health and mental capacity have indignantly turned their backs upon their villages and farm cottages, and have sought more favourable conditions of life in large towns or in foreign emigration. Only the very dregs of the labouring population are left behind in many districts; and these poor drudges, by the irony of fate, are now becoming masters of the situation. They demand higher wages than even the best men have long been accustomed to receive, and the farmers, on the verge of ruin, have no choice but to yield. It thus happens that the punishment of short-sightedness is the failure of service when service is needed, and the unavoidable necessity of paying much more for poor service than would willingly have been accepted for good service if the concession had been made in time.

The same rules must, and do, apply to the medical profession, for political economy takes no account whatever of education or social condition. What it takes account of is the fact of supply and demand. It is certain that at the present time there are too many medical men in the country. As a result of this the public are able to force down the fees of the doctor. But the further inevitable consequence of that will be that the more capable among the younger practitioners will desert medicine, the clever boys will shun it as an unworthy occupation, and the average general practitioner will gradually revert to the position and capability of the old village apothecary. There will be yet one further result of this degrading process; the medical profession will no longer consist of cultured and capable men, but of half-educated and self-asserting pretenders; and those men, by the inevitable operation of the laws of political economy, will be able to charge twice as much for their comparatively worthless services as the well-trained doctor of to-day can now charge for his rational experience and highly-trained skill.

The defender of sixpence a quarter mixes up and hopelessly confounds two very different questions. He says in effect that the doctor must attend the poor, whether he desires it or not; and that it is better for him to be paid a little than to be paid nothing. That is the same as saying that it is better to cross a river in a boat with its side staved in than to attempt to swim it. For our part, we prefer the swim. The attempt to cross in the mutilated boat can end in nothing but the most complete and ridiculous failure. But as a matter of fact, is the doctor compelled to attend the very poor? This question may be answered by asking one or two others. Is the baker compelled to feed the poor, the tailor to clothe them, or the proprietor to house them? No one will deny that food, clothing, and shelter are as fundamentally necessary as medicine and attendance. In cases where the poor are so very poor that they cannot provide food, clothing, and shelter, those things are provided for them by the local authorities. Medicine and medical attendance are provided for them in exactly the same circumstances. It is as certain as certain can be, to those who know, that it is not the poverty of the poor at all that causes the lowering of medical fees, but the senseless stupidity and detestable professional jealousy of medical men. Three doctors in every six would infinitely rather attend a hundred patients for starvation fees than see a single one go to a rival practitioner. Such doctors often make acquaintance with poverty, debt, and the Bankruptcy Court; and right well are they served.

It is surely but a commonplace to say that all work which is done for pay should be paid for according to its value. Work which is done as a charity should be done generously and freely, and without thought of pay. Work which is underpaid is unsatisfactory work; it degrades both the worker and the payer. Similarly, charity which demands even the very slightest return is unsatisfactory as charity; it pleases neither the giver nor the receiver. Work, we repeat, must be paid for, and paid for properly; charity is essentially a gift, and cannot be too free. To mix paid work and charity together, and to confound them as if they were necessarily part of each other is one of the most unbusiness-like proceedings in the world. No man of the world would ever think of it. It is only possible to old women and muddle-headed doctors.

The whole question resolves itself into this: Can a medical man give honest and competent attendance and medicine to a child for sixpence a quarter? Most emphatically he can not! But it is argued that "the numbers make it pay." Can, then, a medical man give attendance and medicine to a hundred children for sixpence a-quarter each; that is, for two pounds ten shillings the lot? Does not the absurdity come out much more strongly when the hundred are considered than when attention is confined to the one? The whole thing is entirely and absolutely preposterous.

But if a medical man cannot give honest and competent service and good medicine to a child for sixpence a-quarter, or to a hundred children for two pound ten, then he must give dishonest and incompetent services, and worthless medicine. And if he gives dishonest and incompetent services and worthless medicine, is he not a dishonest and worthless man? And if, whilst giving dishonest and incompetent services and worthless medicine, he is professing to give services that are honest and competent and medicine that is suitable and good, is he not practising a fraud? And if a man be fraudulent, is he not a scoundrel? And if he be a scoundrel, may it not be right and proper, and in every way necessary and desirable, to tell him so in good round terms, whether he be a doctor, a duke, an "Artful Dodger," or a "Mr. William Sykes"?

The sixpenny doctor thinks that the writer of our former article could not be a doctor, or he would not have spoken against members of his own craft. Did our critic ever hear of a man who was jealous for the honour of his own house? Does not every man of honour feel that his first duty is to act honourably himself; his next to see that his family acts honourably; and his next to do his utmost to preserve the honourable character of his profession? It is because the present writer is a doctor that he criticises doctors and medicine. If he were a lawyer he would criticise lawyers and law, and a precious tough task he would have—a task that Hercules himself might shrink from. In sober truth, sixpenny doctors and all their tribe are overwhelming the medical profession in vulgar and ignominious ruin. The question is so serious that every honest and intelligent practitioner ought to take it up. The public, too, in self-defence, should resist the degradation of medicine. It is as necessary to have rational and efficient medicine as to have reasonable religion and just law. But we cannot have any single one of these unspeakable blessings without paying for it.

